# Differences in subjective age by filial caregiving status among US adults in mid and later life

**DOI:** 10.1093/geroni/igab046.2956

**Published:** 2021-12-17

**Authors:** Hyojin Choi, Kristin Litzelman

**Affiliations:** 1 University of Wisconsin--Madison, Madison, Wisconsin, United States; 2 University of Wisconsin-Madison, Madison, Wisconsin, United States

## Abstract

Subjective age is an important indicator of age identity and is associated with both psychological and physical well-being. Previous studies have revealed that older adults who feel younger than their chronological age show better health status, better life satisfaction, and less risk of mortality. Considerable evidence shows that stress contributes to feeling older than one’s chronological age. Given the fact that taking a caregiving role involves stress, it is expected that caregiving might accelerate subjective aging. This study examined the association between the stressor of caregiving and subjective age in mid and later life. Data were drawn from the Health and Retirement Study in 2014 and 2016. Participants aged 50 years and over (n=1,087) were identified according to adult-child caregiver status at across the two waves: those who provided care consecutively (long-term caregivers), those who became caregivers in 2016 (new caregivers), those who were no longer providing care in 2016 (recent caregivers), or those who did not report providing care in both 2014 and 2016 (non-caregivers). Linear regression analysis showed that new caregivers reported feeling older than their chronological age compared to non-caregivers. However, long-term or recent caregivers did not show significant differences in subjective age compared to non-caregivers. The finding is consistent with the stress process theory and adaptation hypothesis. Although the onset of caregiving stress may accelerate subjective aging, this deleterious effect may decrease over time due to family caregivers’ adaptability. Future research will examine the role of support, resilience and mastery in this pathway.

